# Influence of depression on genetic predisposition to type 2 diabetes in a multiethnic longitudinal study

**DOI:** 10.1038/s41598-017-01406-y

**Published:** 2017-05-09

**Authors:** Sophiya Garasia, Zainab Samaan, Hertzel C. Gerstein, James C. Engert, Viswanathan Mohan, Rafael Diaz, Sonia S. Anand, David Meyre

**Affiliations:** 1 Department of Clinical Epidemiology and Biostatistics, McMaster University, Hamilton, Ontario Canada; 2 Department of Psychiatry and Behavioral Neurosciences, McMaster University, Hamilton, ON Canada; 3 Population Health Research Institute, McMaster University and Hamilton Health Sciences, Hamilton General Hospital, Hamilton, Ontario Canada; 4 Department of Medicine, McMaster University, Hamilton, Ontario Canada; 5 Department of Medicine, McGill University, Montreal, QC Canada; 6 Department of Human Genetics, McGill University, Montreal, QC Canada; 7 Research Institute of the McGill University Health Centre, Montreal, QC Canada; 8 Madras Diabetes Research Foundation, Chennai, India; 9 ECLA Academic Research Organization, Rosario, Argentina; 10 Department of Pathology and Molecular Medicine, McMaster University, Hamilton, Ontario Canada

## Abstract

We assessed the association between depression status and prevalent and incident type 2 diabetes (T2D) as well as the interaction between depression and a genetic risk score (GS) based on 20 T2D single-nucleotide polymorphisms (SNPs) in a multi-ethnic longitudinal study. We studied 17,375 participants at risk for dysglycemia. All participants had genotypic and phenotypic data collected at baseline and 9,930 participants were followed-up for a median of 3.3 years. Normal glucose tolerance (NGT), impaired fasting glucose (IFG)/impaired glucose tolerance (IGT) and T2D statuses were determined using an oral glucose tolerance test and the 2003 American Diabetes Association criteria. Depression was diagnosed at baseline using Diagnostic and Statistical Manual of Mental Disorders, Fourth Edition (DSM IV). Multivariate logistic regression models were adjusted for age, sex, ethnicity and body-mass index and an interaction term GS X depression was added to the model. After appropriate Bonferroni correction, no significant association between depression and T2D-related traits (IFG/IGT, T2D and dysglycemia), and no significant interaction between the GS and depression status was observed at baseline or follow-up. Our longitudinal data do not support an association between depression and abnormal glycemic status. Moreover, depression does not modify the effect of T2D predisposing gene variants.

## Introduction

Type 2 diabetes (T2D) is a major public health concern as it has turned into a global epidemic^1^. According to the International Diabetes Federation, 382 million individuals had diabetes in 2013, and this number is projected to reach 592 million in 2035 (http://www.idf.org/). T2D is a lifelong chronic disease that is difficult to treat despite a plethora of available treatments^2^. In that context, an improved understanding of the etiology of T2D is urgently needed to assist future T2D prevention programs^3, 4^.

Two meta-analyses have showed that depression increases the risk of incident T2D by 37–60%^5, 6^. Disturbances in insulin resistance, glucocorticoid signaling and inflammation, adipokine synthesis and secretion, and mitochondrial respiration have been proposed to link depression and subsequent development of T2D^7, 8^. As such, depression has been referred as ‘metabolic syndrome II’ in the literature^7^. In addition, weight gain is a side effect of anti-depressant medications and may represent an additional risk factor for T2D^9^. However, despite these observations, the biological mechanisms that explain how depression leads to T2D are still unclear and require further research.

Evidence that the association between depression and T2D results from pleiotropic predisposing genes is sparse and conflicting^10, 11^. An alternative explanation may be that depression amplifies the effect of T2D genes in predisposed populations. Indirectly supporting this view, Rivera *et al*. found an interaction between the major genetic contributor to obesity *FTO* and depression on body mass index (BMI) in European populations^12^. However, no interaction study between T2D predisposing gene variants and depression has been reported so far. We used a genetic risk score (GS) based on 20 SNPs identified by genome-wide association studies for T2D to investigate the modifying effect of depression on the genetic predisposition to T2D-related traits in a multi-ethnic longitudinal study.

## Results

### Baseline and follow-up characteristics of depression in cases and controls

Table 1 shows participants characteristics at baseline and follow-up by depression status in the EpiDREAM study. At baseline, a total of 17,375 individuals were included from which 3203 (18.4%) were diagnosed as depressed cases and 14,172 individuals (81.4%) were not. Sixty-one percent of the study participants were female and 75.8% of depressed cases were female. Two thousand five hundred and fifty-eight (14.7%) study participants had T2D at baseline while the majority was classified as having NGT (42.8%) or IFG or IGT (42.5%). Four hundred and twenty-five patients with depression were also diagnosed with T2D (13.3%). Depressed cases were on average two years younger and had a 1.75 kg/m^2^ higher BMI than non-depressed controls. In terms of ethnic distribution, South Asians and East Asians reported less depression compared to Latin Americans and Native North Americans (Table 1 and Supplementary Table 2). One hundred and seventy-nine (9.7%) participants in the depression group at baseline developed incident IFG/IGT whereas 792 (9.8%) non-depressed controls converted from NGT to IFG/IGT at follow-up. Two hundred and sixty-two (14.2%) participants who reported depression at baseline and 1,008 (12.5%) in the non-depressed group had developed incident T2D at follow-up. The overall distribution of glycemic status was similar in the depression case and control groups.

**Table 1:** Baseline and follow-up characteristics by depression status in the EpiDREAM study.

Traits	Categories	All	Depression Case	Non Depressed Control	*P*-value^c^
**Baseline Characteristics**					
Sex n (%)	Female	10598 (61.0)	2428 (75.8)	8170 (57.6)	<0.001
	Male	6777 (39.0)	775 (24.2)	6002 (42.4)	
Glycemic status n (%)	NGT	7431 (42.8)	1439 (44.9)	5992 (42.3)	0.006
	IFG OR IGT^b^	7386 (42.5)	1339 (41.8)	6047 (42.6)	
	Type 2 diabetes	2558 (14.7)	425 (13.3)	2133 (15.0)	
Age Mean ± SD (n)^a^		52.66 ± 11.37 (17375)	51.08 ± 10.55 (3203)	53.01 ± 11.52 (14172)	<0.001
BMI Mean ± SD (n)^a^		30.16 ± 6.22 (17369)	31.59 ± 6.78 (3201)	29.84 ± 6.04 (14168)	<0.001
Ethnicity n (%)	South Asian	2754 (15.8)	191 (6.0)	2563 (18.1)	<0.001
	East Asian	225 (1.3)	23 (0.7)	202 (1.4)	
	European	9363 (53.9)	1748 (54.6)	7615 (53.7)	
	African	1247 (7.2)	225 (7.0)	1022 (7.2)	
	Latin American	3287 (18.9)	914 (28.5)	2373 (16.8)	
	Native North American	499 (2.9)	102 (3.2)	397 (2.8)	
	Total	17375 (100)	3203 (100)	14172 (100)	
**Follow-up Characteristics**					
Glycemic status n (%)	^d^NGT at baseline and follow-up	2,554 (25.7)	476 (25.8)	2,078 (25.7)	0.21
	^d^Incident IFG/IGT (%)	971 (9.8)	179 (9.7)	792 (9.8)	
	^d^Incident T2D N (%)	1,270 (12.8)	262 (14.2)	1,008 (12.5)	
	Other participants followed-up (%)	5,135 (51.7)	928 (50.3)	4,207 (52.0)	

^a^SD = standard deviation. ^b^NGT = normal glucose tolerance; IFG = impaired fasting glucose; IGT = impaired glucose tolerance. ^c^ *P* are reported for unadjusted univariable analyses (Chi-square or Student T-tests. ^d^Glycemic status based on OGTT.

### Association between baseline depression and T2D-related traits at baseline and follow-up

Using a logistic regression model adjusted for sex, age, BMI and ethnicity, depression was not associated with prevalent IFG/IGT (OR = 0.97 [0.89–1.06], *P* = 0.51), T2D (OR = 1.00 [0.88–1.13], *P* = 0.96) or dysglycemia (OR = 0.98 [0.90–1.06], *P* = 0.59) (Table 2). No association was found between baseline depression and incident IFG/IGT (OR = 0.97 [0.80–1.19], *P* = 0.79), T2D (OR = 1.22 [1.01–1.47], *P* = 0.04) or dysglycemia (OR = 1.09 [0.94–1.28], *P* = 0.26) after appropriate Bonferroni correction (P_corrected_ = 0.0083 (0.05/6), Table 2).

**Table 2:** Association between depression and T2D-related traits at baseline and follow-up.

Status	OR	95% CI	*P*-value
Baseline			
NGT vs. IFG/IGT	0.97	0.89–1.06	0.51
NGT vs. T2D	1.00	0.88–1.13	0.96
NGT vs. dysglycemia	0.98	0.90–1.06	0.59
Follow-up			
NGT vs. IFG/IGT	0.97	0.80–1.19	0.79
NGT vs. T2D	1.22	1.01–1.47	0.04
NGT vs. dysglycemia	1.09	0.94–1.28	0.26

Logistic regression models were adjusted for sex, age, BMI and ethnicity.

### Interaction between the genotype score and depression on T2D-related traits at baseline and follow-up

Using a logistic regression model adjusted for sex, age, BMI and ethnicity, the GS was associated with IFG/IGT, T2D and dysglycemia statuses at baseline (OR = 1.07 [1.06–1.08] *P* = 5.9 × 10^−26^, OR = 1.11 [1.09–1.13] *P* = 5.9 × 10^−31^, OR = 1.08 [1.07–1.09] *P* = 4.7 × 10^−38^, respectively) and follow-up (OR = 1.05 [1.03–1.08] *P* = 1.7 × 10^−4^, OR = 1.14 [1.11–1.17] *P* = 2.1 × 10^−20^, OR = 1.10 [1.07–1.12] *P* = 4.0 × 10^−16^, respectively, Table 3). No significant interaction between the GS and depression status on T2D-related traits was observed at baseline and follow-up after appropriate Bonferroni correction (P_corrected_ = 0.0083 (0.05/6), (Table 3)).

**Table 3:** Main effect and interaction of GS with depression status on T2D-related-traits at baseline and follow-up.

	IFG/IGT status		T2D status		Dysglycemia status	
	Main Effect	Interaction	Main Effect	Interaction	Main Effect	Interaction
Gene score at Baseline	**1.07**[**1.06**–**1.08**] (**5.9** × **10** ^**−26**^)	1.01[0.98–1.04] (0.55)	**1.11** [**1.09**–**1.13**] (**5.9** × **10** ^**−31**^)	**1.052** [**1.01**–**1.10**] (**0.026**)	**1.08**[**1.07**–**1.09**] (**4.7** × **10** ^**−38**^)	1.02 [0.99–1.05] (0.16)
Gene score at Follow-up	**1.05**[**1.03**–**1.08**] (**1.7** × **10** ^**−4**^)	1.04[0.97–1.12] (0.25)	**1.14**[**1.11**–**1.17**] (**2.1** × **10** ^**−20**^)	1.01[0.94–1.07] (0.86)	**1.10**[**1.07**–**1.12**] (**4.0** × **10** ^**−16**^)	1.02[0.96–1.07] (0.53)

Logistic regression models adjusted for sex, age, BMI and ethnicity.

## Discussion

We investigated the association between depression at baseline and prevalent and incident abnormal glucose homeostasis in a longitudinal international study that enrolled up to 17,375 subjects from six ethnic groups. Our data do not support a direct association between depression and T2D-related traits at the epidemiological level, keeping in mind that our power calculations do not totally exclude the possibility of a subtle but not clinically meaningful association between depression and abnormal glucose homeostasis (Supplementary Figure 2). Studies have shown that many behaviors and physiological changes associated with depression are risk factors for insulin resistance. This includes lower physical activity levels^13^, weight change^14^, and cortisol levels^15^. However, evidence of association between depression and altered glucose homeostasis has been conflicting in epidemiological studies^6, 16^.

We also observed an absence of interaction between a T2D GS and depression on T2D-related traits at baseline and at follow up. These interaction tests add to our recent report of a lack of association of T2D predisposing SNPs with depression status at baseline in EpiDREAM^10^. In contrast, observational and genetic epidemiology studies by us and others have validated the existence of shared biological mechanisms between depression and obesity, an important risk factor for T2D^12, 17–19^. Consistent with this view, the nominal evidence of association observed between baseline depression and incident T2D (OR = 1.22 [1.01–1.47], *P* = 0.04) was strengthened when BMI was removed as a covariate in the model (OR = 1.32 [1.10–1.59], *P* = 0.003). Meta-analyses showing an association between depression status and T2D may have been biased by the absence of adjustment for BMI in most included studies^5, 6^.

Strengths of this study include a large study population including diverse ethnic backgrounds with standardized measures of glycemic and depression statuses. The selection of individuals at risk for dysglycemia increased the power for of the study. In addition, the longitudinal assessment of the glycemic status allows causal inference to be made on the results. Studying the association between depression and glycemic statuses at the epidemiological and genetic level strengthens our conclusions. Limitations of our study include a non-exhaustive list of T2D-associated SNPs that were included in the GS. The ascertainment of the study for individuals at risk for dysglycemia limits the generalization of our results to general populations. We acknowledge that our study was modestly powered for gene by environment interaction studies, especially at follow-up with reduced number of participants, and did not enable the investigation of ethnic-specific associations (Supplementary Figures 2 and 3). In addition the distribution of ethnic groups was not equally represented (Table 1). Our baseline diagnosis for depression is based on the last 12 months and as such does not represent a lifelong depression status. On the other end, a recall bias can possibly be introduced when asking participants about depressive symptoms in the past year. As the questionnaire focuses on major depressive disorder diagnosis, it does not provide a quantitative indication on the severity of the depressive episode. It also does not provide an indication of the duration of the depressive episode. We are also aware that the reliability of the major depressive disorder based on Diagnostic and Statistical Manual of Mental Disorders, Fourth Edition (DSM IV) in our study is not perfect. Every method of diagnosing psychiatric disorders has its shortcomings as the core of psychopathology relies on subjective reporting of symptoms in addition to observable traits during illness episodes. As an illustration, the reliability of diagnosis of depression based on structured clinical interviews for DSM (SCID) has been questioned in literature^20, 21^.

In conclusion, our longitudinal data do not support an association between depression and abnormal glycemic status and genetic predisposition to T2D is not modified by the presence of depression.

## Methods

### Participants

EpiDREAM is a longitudinal study that enrolled 24,872 individuals from 21 countries who were at risk for T2D, including subjects who participated in the DREAM clinical trial^22^. All individuals who were deemed to be at risk for dysglycemia defined by family history, ethnicity and abdominal obesity, between the ages of 18–85 years, were screened using a 75 gram oral glucose tolerance test (OGTT) from July 2001 to August 2, 2003. Detailed methods and description of the study cohort and inclusion/exclusion criteria have been described earlier^23, 24^. We included 17,375 subjects from six ethnic groups (East Asian, South Asian, European, African, Latin American, Native North American) who have both phenotypic and genotypic information available at baseline (Supplementary Figure 1). Self-reported ethnicity has been validated in the 17,375 individuals using the eigenstrat software (http://genepath.med.harvard.edu/~reich/Software.htm). Samples that failed to cluster with individuals of the same self-reported ethnicity were removed. Of these 17,375 individuals, 9,930 have been prospectively followed for a median of 3.3 years. The EpiDREAM and DREAM studies were approved by local ethics committees. All experimental protocols were approved by McMaster University and were performed in accordance with relevant guidelines and regulations of McMaster University. Written informed consent was obtained from each subject prior to participation in the EpiDREAM or DREAM studies, in accordance with the Declaration of Helsinki.

### Genotyping

DNA was successfully extracted from buffy coats in 19,498 participants of the EpiDREAM study using the Gentra System (Supplementary Figure 1). Genotyping was performed using the Illumina cardiovascular gene-centric bead chip ITMAT Broad Care (IBC) array^25^. Genotyping was performed at the McGill University and Genome Quebec Innovation Centre using the Illumina Bead Studio genotyping module, version 3.2. We established a list of SNPs that reached genome-wide significance (P < 5 × 10^−8^) for association with T2D status in populations of European ancestry. We used three different strategies to optimize the SNP selection procedure using a key word search on i) the National Human Genome Research Institute (NHGRI) GWAS Catalog (www.genome.gov/gwastudies/) ii) the HuGE Navigator GWAS Integrator (www.hugenavigator.net/HuGENavigator/gWAHitStartPage.do) iii) the PubMed database (www.ncbi.nlm.nih.gov/pubmed). Using this strategy, we found 93 independent SNPs associated with T2D in literature. From this list, 20 autosomal SNPs were available on versions 1 and 2 of the IBC 50 K SNP array (Supplementary Table 1), as reported in our previous paper^10^. We double checked the availability of proxies using SNAP and their chromosomal position in the Illumina data file, as previously reported by our team^26^. We did not find additional SNPs for inclusion. The 20 SNPs include: rs1260326 (*GCKR*), rs2943634 (*IRS1*), rs1801282 (*PPARγ2*), rs1470579 (*IGF2BP2*), rs1801214 (*WFS1*), rs7754840 (*CDKAL1*), rs1799884 (*GCK*), rs13266634 (*SLC30A8*), rs2383208 (*CDKN2A/B*), rs5015480 (*HHEX*), rs7903146 (*TCF7L2*), rs231362 (*KCNQ1*), rs2283228 (*KCNQ1*), rs5219 (*KCNJ11*), rs10830963 (*MTNR1B*), rs4430796 (*HNF1B*), rs12454712 (*BCL2*), rs16996148 (*GATAD2A*), rs8108269 (*GIPR*), and rs1884614 (*HNF4A*). SNPs obeyed Hardy-Weinberg equilibrium (HWE) within each ethnic group in the overall sample as well as in the depressed and non-depressed subgroups (*P* ≥ 0.00014 (0.05/(6 × 20 × 3)), Supplementary Table 1), with the exception of one SNP. We detected a significant departure from HWE for rs1884614 (*HNF4A*) in the Latin American group, but not in the other ethnic groups (Supplementary Table 1). This suggests that the HWE identified in the Latin American group for this SNP likely did not result from genotyping errors but was the consequence of the admixed nature of this population. Therefore, all the SNPs were included in the study. The call rate for each of the 20 SNPs was between 99.33 and 100% (Supplementary Table 1).

### Phenotyping

Demographic data as well as direct anthropometric measurements were obtained from study participants using a standardized protocol. Height (m) and weight (kg) were measured in clinical centers by trained staff. BMI was calculated as weight in kilograms (kg) divided by height in meters (m) squared. The 2003 ADA criteria were used to classify participants as having normal glucose tolerance (NGT), impaired fasting glucose (IFG), impaired glucose tolerance (IGT), or T2D at baseline, following an oral glucose tolerance test (OGTT). Normoglycemia was defined as a fasting plasma glucose <5.6 mmol/L, IFG was defined as a fasting plasma glucose of 5.6 to 6.9 mmol/L, IGT was defined as a fasting plasma glucose below 7.0 mmol/L and a 2-h glucose between 7.8 and 11.0 mmol/L, and diabetes was defined if either the fasting plasma glucose was ≥7.0 mmol/L or the 2-h glucose was ≥11.1 mmol/L^27^. At follow-up, glycemic status was ascertained using i) an OGTT conducted at the final exam; or ii) a diagnosis made by a participant’s physician during the course of the study, confirmed by participant’s use of an oral hypoglycaemic agent, and an OGTT. Subjects with IFG, IGT or T2D were considered as having dysglycemia.

The assessment of a major depressive episode in the past 12 months was performed at baseline. Interviews were conducted face to face by research personnel who were not mental health specialists but were research assistants trained in the study procedures and administration of the questionnaires, including inter rater reliability to calibrate the training. A structured case report form was completed in which participants were asked: “During the past 12 months, was there ever a time when you felt sad, blue, or depressed for 2 weeks or more in a row?”. If the participant answered yes, then the following yes or no questions were asked: (a) Lose interest in most things like hobbies, work or activities that usually give you pleasure?; (b) Feel tired or low on energy?; (c) Gain or lose weight?; d) Have more trouble falling asleep than you usually do?; (e) Have more trouble concentrating than usual?; (f) Think a lot about death (either your own, someone else’s, or death in general)?; (g) Feel down on yourself, no good or worthless? These questions were compiled to fulfil DSM IV^28^ diagnostic criteria for a depressive episode. A depressive episode was considered present if the individual had five or more of the above symptoms including depressed mood that were present for at least 2 weeks nearly every day for most of the day in the last 12 months.

### Statistical analyses

All statistical analyses were performed using the SPSS 14.0 software. The comparison of baseline characteristics between depression cases and controls was done using T-tests or Chi-square tests. We coded genotypes as 0, 1 and 2, depending on the number of copies of the T2D risk alleles. A GS was calculated by summing the alleles of the 20 T2D predisposing SNPs. We used an unweighted GS as recommended by Dudbridge^29^. Missing genotype values were imputed using the method of the mean for each SNP individually in each ethnicity separately using the arithmetic average of the coded genotypes observed for all the successfully genotyped individuals. The association of GS with T2D-related traits was tested using a logistic regression model. Regression models were adjusted for age, sex, BMI, ethnicity/population stratification and depression and a GS by depression status interaction term was added to the model. Ethnicity and population stratification were accounted for using the first 10 axes of variation from Principal Components Analysis, determined using EIGENSTRAT^30^. Applying a Bonferroni corrected P-value across all the outcomes reduces the chance of making type I errors, but increases the chance of making type II errors. Therefore, we applied a separate Bonferroni correction to each research question^31^. A P-value of less than 0.0083 was considered statistically significant when analyses of associations between baseline depression and T2D-related traits at baseline and follow-up were performed as well as when interactions between baseline depression, and the GS on T2D-related traits at baseline and follow-up were performed (6 analyses in total).

## Electronic supplementary material

online supplementary material
