# Supplementary material for: Influence of depression on genetic predisposition to type 2 diabetes in a multiethnic longitudinal study
Source: Sci Rep. 2017 May 9;7:1629. doi: 10.1038/s41598-017-01406-y (PMC5431642; doi:10.1038/s41598-017-01406-y)
Supplement: Supplementary file 1 — online supplementary material [file 41598_2017_1406_MOESM1_ESM.pdf]

# Influence of depression on genetic predisposition to type 2 diabetes in a multiethnic longitudinal study

**Sophiya Garasia[1,10], Zainab Samaan[1,2,3,10], Hertzel C. Gerstein[1,3,4], James C. Engert[5,6], Viswanathan Mohan[7], Rafael Diaz[8], Sonia S. Anand[1,3,4], David Meyre[1,3,9*]**

[1]Department of Clinical Epidemiology and Biostatistics, McMaster University, Hamilton, Ontario, Canada; [2]Department of Psychiatry and Behavioral Neurosciences, McMaster University, Hamilton, ON, Canada; [3]Population Health Research Institute, McMaster University and Hamilton Health Sciences, Hamilton General Hospital, Hamilton, Ontario, Canada; [4]Department of Medicine, McMaster University, Hamilton, Ontario, Canada; [5]Department of Medicine, McGill University, Montreal, QC, Canada; [6]Department of Human Genetics, McGill University, Montreal, QC; Research Institute of the McGill University Health Centre, Montreal, QC, Canada; [7]Madras Diabetes Research Foundation, Chennai, India; [8]ECLA Academic Research Organization, Rosario, Argentina; [9]Department of Pathology and Molecular Medicine, McMaster University, Hamilton, Ontario, Canada; [10] Shared first authorship.

**Supplementary Table S1.** Genotype distributions of 20 T2D predisposing SNPs in the EpiDREAM Study.

| | | | | **All** | | | | | | **Depression cases** | | | | | | **Depression controls** | | | | | |
|---|---|---|---|---|---|---|---|---|---|---|---|---|---|---|---|---|---|---|---|---|---|
| Ethnicity | Major allele | Minor allele | T2D risk allele | Genotype count | | | T2D risk allele frequency | Call rate (%) | HWE *P*-value | Genotype count | | | T2D risk allele frequency | Call rate (%) | HWE *P*-value | Genotype count | | | T2D risk allele frequency | Call rate (%) | HWE *P*-value |
| Rs1260326 – ***GCKR*** | | | | | | | | | | | | | | | | | | | | | |
| | | | | CC | CT | TT | | | | CC | CT | TT | | | | CC | CT | TT | | | |
| South Asian | C | T | C | 1712 | 927 | 114 | 0.790 | 99.96 | 0.411 | 128 | 60 | 3 | 0.827 | 100 | 0.171 | 1584 | 867 | 111 | 0.787 | 99.96 | 0.577 |
| East Asian | | | | 73 | 108 | 44 | 0.564 | 100 | 0.721 | 10 | 8 | 5 | 0.609 | 100 | 0.196 | 63 | 100 | 39 | 0.559 | 100 | 0.952 |
| European | | | | 3128 | 4575 | 1658 | 0.579 | 99.98 | 0.833 | 554 | 877 | 317 | 0.568 | 100 | 0.353 | 2574 | 3698 | 1341 | 0.597 | 99.97 | 0.004 |
| African | | | | 955 | 271 | 21 | 0.875 | 100 | 0.726 | 166 | 53 | 6 | 0.856 | 100 | 0.481 | 789 | 218 | 15 | 0.879 | 100 | 0.989 |
| Latin American | | | | 1291 | 1482 | 513 | 0.618 | 99.97 | 0.011 | 378 | 381 | 155 | 0.622 | 100 | $6.0x10^{-4}$ | 913 | 1101 | 358 | 0.617 | 99.96 | 0.393 |
| Native | | | | 236 | 211 | 52 | 0.684 | 100 | 0.635 | 43 | 49 | 10 | 0.662 | 100 | 0.460 | 193 | 162 | 42 | 0.690 | 100 | 0.361 |
| Total | | | | 7395 | 7574 | 2402 | 0.644 | 99.98 | N.A. | 1279 | 1428 | 496 | 0.622 | 100 | N.A. | 6116 | 6146 | 1906 | 0.649 | 99.97 | N.A. |
| Rs2943634 – *IRS1* | | | | | | | | | | | | | | | | | | | | | |
| | | | | CC | CT | TT | | | | CC | CT | TT | | | | CC | CT | TT | | | |
| South Asian | C | T | C | 1732 | 900 | 117 | 0.794 | 99.82 | 0.995 | 124 | 58 | 9 | 0.801 | 100 | 0.513 | 1608 | 842 | 108 | 0.793 | 99.81 | 0.866 |
| East Asian | | | | 186 | 38 | 1 | 0.911 | 100 | 0.522 | 19 | 4 | 0 | 0.913 | 100 | 0.648 | 167 | 34 | 1 | 0.911 | 100 | 0.600 |
| European | | | | 4162 | 4142 | 1046 | 0.667 | 99.86 | 0.749 | 784 | 754 | 207 | 0.665 | 99.83 | 0.214 | 3378 | 3388 | 839 | 0.667 | 99.87 | 0.810 |
| African | | | | 236 | 611 | 398 | 0.435 | 99.84 | 0.956 | 34 | 124 | 66 | 0.429 | 99.56 | 0.051 | 202 | 487 | 332 | 0.436 | 99.90 | 0.333 |
| Latin American | | | | 1762 | 1245 | 271 | 0.727 | 99.73 | 0.016 | 517 | 321 | 70 | 0.746 | 99.34 | 0.044 | 1245 | 924 | 201 | 0.720 | 99.87 | 0.113 |
| Native | | | | 320 | 153 | 26 | 0.795 | 100 | 0.175 | 74 | 23 | 5 | 0.838 | 100 | 0.089 | 246 | 130 | 21 | 0.783 | 100 | 0.483 |
| Total | | | | 8398 | 7089 | 1859 | 0.688 | 99.83 | N.A. | 1552 | 1284 | 357 | 0.687 | 99.69 | N.A. | 6846 | 5805 | 1502 | 0.689 | 99.87 | N.A. |
| Rs1801282 – *PPARG* | | | | | | | | | | | | | | | | | | | | | |
| | | | | CC | CG | GG | | | | CC | CG | GG | | | | CC | CG | GG | | | |
| South Asian | C | G | C | 2143 | 568 | 43 | 0.881 | 100 | 0.447 | 147 | 41 | 3 | 0.877 | 100 | 0.942 | 1996 | 527 | 40 | 0.882 | **100** | 0.442 |
| East Asian | | | | 203 | 22 | 0 | 0.951 | 100 | 0.441 | 21 | 2 | 0 | 0.957 | 100 | 0.827 | 182 | 20 | 0 | 0.950 | 100 | 0.459 |
| European | | | | 7273 | 1964 | 126 | 0.882 | 100 | 0.612 | 1380 | 343 | 25 | 0.888 | 100 | 0.485 | 5893 | 1621 | 101 | 0.880 | 100 | 0.378 |
| African | | | | 1204 | 43 | 0 | 0.983 | 100 | 0.536 | 219 | 6 | 0 | 0.987 | 100 | 0.839 | 985 | 37 | 0 | 0.982 | 100 | 0.556 |
| Latin American | | | | 2364 | 610 | 42 | 0.885 | 91.76 | 0.710 | 726 | 172 | 16 | 0.940 | 100 | 0.123 | 1908 | 438 | 26 | 0.897 | 99.96 | 0.878 |
| Native | | | | 399 | 95 | 5 | 0.895 | 100 | 0.803 | 80 | 20 | 2 | 0.882 | 100 | 0.575 | 319 | 75 | 3 | 0.898 | 100 | 0.535 |
| Total | | | | 13856 | 3302 | 216 | 0.893 | 99.99 | N.A. | 2573 | 584 | 46 | 0.894 | 100 | N.A. | 11283 | 2718 | 170 | 0.450 | 99.99 | N.A. |
| Rs1470579 – *IGF2BP2* | | | | | | | | | | | | | | | | | | | | | |
| | | | | AA | AC | CC | | | | AA | AC | CC | | | | AA | AC | CC | | | |
| South Asian | A | C | C | 765 | 1308 | 674 | 0.483 | 99.75 | 0.015 | 52 | 91 | 48 | 0.490 | 100 | 0.519 | 713 | 1217 | 626 | 0.483 | 99.73 | 0.018 |
| East Asian | | | | 119 | 89 | 17 | 0.273 | 100 | 0.949 | 9 | 12 | 2 | 0.348 | 100 | 0.472 | 110 | 77 | 15 | 0.265 | 100 | 0.764 |
| European | | | | 4275 | 4085 | 1002 | 0.528 | 99.99 | 0.574 | 787 | 761 | 200 | 0.332 | 100 | 0.436 | 3488 | 3324 | 802 | 0.324 | 99.99 | 0.809 |
| African | | | | 72 | 431 | 743 | 0.769 | 99.92 | 0.366 | 7 | 74 | 144 | 0.804 | 100 | 0.497 | 65 | 357 | 599 | 0.762 | 99.90 | 0.233 |
| Latin American | | | | 1517 | 1434 | 333 | 0.639 | 99.91 | 0.827 | 417 | 407 | 89 | 0.641 | 99.89 | 0.474 | 1100 | 1027 | 244 | 0.319 | 99.92 | 0.851 |
| Native | | | | 304 | 170 | 25 | 0.220 | 100 | 0.845 | 59 | 38 | 5 | 0.235 | 100 | 0.722 | 245 | 132 | 20 | 0.217 | 100 | 0.685 |

| | | | | | | | | | | | | | | | | | | | | | |
|---|---|---|---|---|---|---|---|---|---|---|---|---|---|---|---|---|---|---|---|---|---|
| Total | | | | 7052 | 7517 | 2794 | 0.378 | 99.93 | N.A. | 1331 | 1383 | 488 | 0.737 | 99.97 | N.A. | 5721 | 6134 | 2306 | 0.759 | 99.92 | N.A. |
| Rs1801214 – *WFS1* | | | | | | | | | | | | | | | | | | | | | |
| | | | | TT | CT | CC | | | | TT | CT | CC | | | | TT | CT | CC | | | |
| South Asian | T | C | T | 1542 | 1020 | 191 | 0.745 | 99.96 | 0.204 | 116 | 66 | 9 | 0.780 | 100 | 0.921 | 1426 | 954 | 182 | 0.743 | 99.96 | 0.196 |
| East Asian | | | | 189 | 32 | 4 | 0.911 | 100 | 0.067 | 20 | 2 | 1 | 0.913 | 100 | 0.030 | 169 | 30 | 3 | 0.911 | 100 | 0.226 |
| European | | | | 3467 | 4517 | 1377 | 0.612 | 99.98 | 0.123 | 655 | 822 | 269 | 0.611 | 99.89 | 0.675 | 2812 | 3695 | 1108 | 0.612 | 100 | 0.059 |
| African | | | | 604 | 520 | 123 | 0.693 | 100 | 0.460 | 115 | 84 | 26 | 0.698 | 100 | 0.085 | 489 | 436 | 97 | 0.640 | 100 | 0.990 |
| Latin American | | | | 1482 | 1430 | 374 | 0.669 | 99.97 | 0.307 | 422 | 397 | 95 | 0.679 | 100 | 0.909 | 1060 | 1033 | 279 | 0.665 | 99.96 | 0.261 |
| Native | | | | 223 | 203 | 73 | 0.650 | 100 | 0.015 | 42 | 42 | 18 | 0.618 | 100 | 0.195 | 181 | 161 | 55 | 0.659 | 100 | 0.041 |
| Total | | | | 7507 | 7722 | 2142 | 0.654 | 99.98 | N.A. | 1370 | 1413 | 418 | 0.649 | 99.94 | N.A. | 6137 | 6309 | 1724 | 0.655 | 82.52 | N.A. |
| Rs7754840 – *CDKAL1* | | | | | | | | | | | | | | | | | | | | | |
| | | | | GG | GC | CC | | | | GG | GC | CC | | | | GG | GC | CC | | | |
| South Asian | G | C | C | 1579 | 999 | 175 | 0.245 | 99.96 | 0.315 | 110 | 65 | 16 | 0.254 | 100 | 0.159 | 1469 | 934 | 159 | 0.244 | 99.96 | 0.518 |
| East Asian | | | | 99 | 92 | 34 | 0.711 | 100 | 0.106 | 13 | 10 | 0 | 0.217 | 100 | 0.182 | 86 | 82 | 34 | 0.371 | 100 | 0.064 |
| European | | | | 4224 | 4085 | 1054 | 0.331 | 100 | 0.162 | 782 | 789 | 177 | 0.327 | 100 | 0.284 | 3442 | 3296 | 877 | 0.332 | 100 | 0.040 |
| African | | | | 228 | 612 | 407 | 0.573 | 100 | 0.938 | 43 | 108 | 74 | 0.569 | 100 | 0.648 | 185 | 504 | 333 | 0.572 | 100 | 0.812 |
| Latin American | | | | 1555 | 1377 | 355 | 0.317 | 100 | 0.056 | 422 | 382 | 110 | 0.329 | 100 | 0.103 | 1133 | 995 | 245 | 0.313 | 100 | 0.226 |
| Native | | | | 206 | 237 | 56 | 0.350 | 100 | 0.323 | 48 | 43 | 11 | 0.319 | 100 | 0.769 | 158 | 194 | 45 | 0.358 | 100 | 0.206 |
| Total | | | | 7891 | 7402 | 2081 | 0.353 | 99.99 | N.A. | 1418 | 1397 | 388 | 0.339 | 100 | N.A. | 6473 | 6005 | 1693 | 0.331 | 99.99 | N.A. |
| Rs1799884 – *GCK* | | | | | | | | | | | | | | | | | | | | | |
| | | | | GG | GA | AA | | | | GG | GA | AA | | | | GG | GA | AA | | | |
| South Asian | G | A | A | 2108 | 596 | 49 | 0.126 | 99.96 | 0.362 | 150 | 36 | 5 | 0.120 | 100 | 0.128 | 1958 | 560 | 44 | 0.126 | 99.96 | 0.588 |
| East Asian | | | | 157 | 62 | 6 | 0.164 | 100 | 0.967 | 16 | 6 | 1 | 0.174 | 100 | 0.659 | 141 | 56 | 5 | 0.163 | 100 | 0.840 |
| European | | | | 6307 | 2743 | 302 | 0.179 | 99.98 | 0.858 | 1169 | 524 | 53 | 0.180 | 99.89 | 0.535 | 5138 | 2219 | 249 | 0.179 | 99.88 | 0.616 |
| African | | | | 829 | 363 | 55 | 0.190 | 100 | 0.061 | 154 | 58 | 13 | 0.187 | 100 | 0.023 | 675 | 305 | 42 | 0.190 | 100 | 0.312 |
| Latin American | | | | 1974 | 1128 | 182 | 0.227 | 99.91 | 0. 213 | 551 | 302 | 59 | 0.230 | 99.78 | 0.047 | 1423 | 826 | 123 | 0.226 | 99.96 | 0.825 |
| Native | | | | 328 | 157 | 14 | 0.185 | 100 | 0.351 | 63 | 37 | 2 | 0.201 | 100 | 0.191 | 265 | 120 | 12 | 0.182 | 100 | 0.721 |
| Total | | | | 11703 | 5049 | 608 | 0.180 | 99.91 | N.A. | 2103 | 963 | 133 | 0.192 | 99.88 | N.A. | 9600 | 4086 | 475 | 0.178 | 99.92 | N.A. |
| Rs13266634 – *SLC30A8* | | | | | | | | | | | | | | | | | | | | | |
| | | | | CC | CT | TT | | | | CC | CT | TT | | | | CC | CT | TT | | | |
| South Asian | C | T | C | 1697 | 926 | 131 | 0.784 | 100 | 0.745 | 116 | 66 | 9 | 0.780 | 100 | 0.921 | 1581 | 860 | 122 | 0.785 | 100 | 0.716 |
| East Asian | | | | 75 | 108 | 41 | 0.576 | 99.56 | 0.846 | 10 | 10 | 3 | 0.652 | 100 | 0.842 | 65 | 98 | 38 | 0.567 | 99.50 | 0.921 |
| European | | | | 4712 | 3841 | 810 | 0.708 | 100 | 0.493 | 876 | 711 | 161 | 0.705 | 100 | 0.335 | 3836 | 3130 | 649 | 0.709 | 100 | 0.768 |
| African | | | | 1043 | 189 | 15 | 0.912 | 100 | 0.057 | 188 | 32 | 5 | 0.907 | 100 | 0.017 | 855 | 157 | 10 | 0.913 | 100 | 0.355 |
| Latin American | | | | 1888 | 1178 | 221 | 0.754 | 100 | 0.044 | 521 | 328 | 65 | 0.749 | 100 | 0.179 | 1367 | 850 | 156 | 0.755 | 100 | 0.127 |
| Native | | | | 272 | 191 | 36 | 0.736 | 100 | 0.756 | 52 | 42 | 8 | 0.716 | 100 | 0.905 | 220 | 149 | 28 | 0.742 | 100 | 0.687 |
| Total | | | | 9687 | 6433 | 1254 | 0.743 | 99.99 | N.A. | 1763 | 1189 | 251 | 0.675 | 100 | N.A. | 7924 | 5244 | 1003 | 0.744 | 99.99 | N.A. |
| Rs2383208– *CDKN2A/B* | | | | | | | | | | | | | | | | | | | | | |
| | | | | AA | AG | GG | | | | AA | AG | GG | | | | AA | AG | GG | | | |
| South Asian | A | G | A | 2000 | 690 | 62 | 0.852 | 99.93 | 0.785 | 143 | 42 | 6 | 0.859 | 100 | 0.193 | 1857 | 648 | 56 | 0.852 | 99.92 | 0.952 |

| | | | | | | | | | | | | | | | | | | | | | |
|---|---|---|---|---|---|---|---|---|---|---|---|---|---|---|---|---|---|---|---|---|---|
| East Asian | | | | 119 | 81 | 25 | 0.709 | 100 | 0.055 | 12 | 10 | 1 | 0.739 | 100 | 0.541 | 107 | 71 | 24 | 0.705 | 100 | 0.028 |
| European | | | | 6465 | 2581 | 314 | 0.829 | 99.97 | 0.005 | 1192 | 499 | 56 | 0.825 | 99.94 | 0.669 | 5273 | 2082 | 258 | 0.829 | 99.97 | 0.003 |
| African | | | | 830 | 372 | 45 | 0.815 | 100 | 0.679 | 158 | 60 | 7 | 0.836 | 100 | 0.657 | 672 | 312 | 38 | 0.810 | 99.96 | 0.811 |
| Latin American | | | | 2249 | 917 | 120 | 0.824 | 99.97 | 0.029 | 621 | 263 | 30 | 0.823 | 100 | 0.739 | 1628 | 654 | 90 | 0.824 | 99.96 | 0.018 |
| Native | | | | 366 | 125 | 8 | 0.859 | 100 | 0.469 | 77 | 25 | 0 | 0.877 | 100 | 0.158 | 289 | 100 | 8 | 0.854 | 100 | 0.849 |
| Total | | | | 12029 | 4766 | 574 | 0.830 | 99.97 | N.A. | 2203 | 899 | 100 | 0.828 | 99.97 | N.A. | 9826 | 3867 | 474 | 0.830 | 99.96 | N.A. |
| Rs5015480– *HHEX* | | | | | | | | | | | | | | | | | | | | | |
| | | | | CC | CT | TT | | | | CC | CT | TT | | | | CC | CT | TT | | | |
| South Asian | C | T | C | 444 | 1360 | 950 | 0.408 | 100 | 0.277 | 32 | 107 | 52 | 0.448 | 382 | 0.066 | 412 | 1253 | 898 | 0.405 | 100 | 0.471 |
| East Asian | | | | 10 | 67 | 148 | 0.193 | 100 | 0.497 | 2 | 8 | 13 | 0.255 | 47 | 0.638 | 8 | 59 | 135 | 0.186 | 100 | 0.629 |
| European | | | | 3319 | 4498 | 1546 | 0.595 | 100 | 0.718 | 608 | 857 | 283 | 0.593 | 3496 | 0.513 | 2711 | 3641 | 1263 | 0.595 | 100 | 0.492 |
| African | | | | 470 | 599 | 178 | 0.617 | 100 | 0.524 | 79 | 115 | 31 | 0.606 | 100 | 0.287 | 391 | 484 | 147 | 0.619 | 100 | 0.888 |
| Latin American | | | | 953 | 1582 | 752 | 0.531 | 100 | 0.054 | 232 | 474 | 208 | 0.513 | 1828 | 0.252 | 721 | 1108 | 544 | 0.537 | 100 | 0.003 |
| Native | | | | 109 | 226 | 164 | 0.445 | 100 | 0.059 | 21 | 43 | 38 | 0.417 | 100 | 0.178 | 88 | 183 | 126 | 0.452 | 100 | 0.166 |
| Total | | | | 5305 | 8332 | 3738 | 0.545 | 100 | N.A. | 974 | 1604 | 625 | 0. 554 | 6406 | N.A. | 4331 | 6728 | 3113 | 0.543 | 100 | N.A. |
| Rs7903146 – *TCF7L2* | | | | | | | | | | | | | | | | | | | | | |
| | | | | CC | CT | TT | | | | CC | CT | TT | | | | CC | CT | TT | | | |
| South Asian | C | T | T | 1312 | 1170 | 272 | 0.311 | 100 | 0.637 | 86 | 84 | 21 | 0.330 | 100 | 0.943 | 1226 | 1086 | 251 | 0.310 | 100 | 0.643 |
| East Asian | | | | 201 | 22 | 2 | 0.058 | 100 | 0.126 | 18 | 4 | 1 | 0.130 | 100 | 0.263 | 183 | 18 | 1 | 0.050 | 100 | 0.450 |
| European | | | | 4512 | 3899 | 952 | 0.310 | 100 | 0.010 | 847 | 731 | 170 | 0.306 | 100 | 0.503 | 3665 | 3168 | 782 | 0.311 | 100 | 0.012 |
| African | | | | 615 | 520 | 112 | 0.298 | 100 | 0.890 | 112 | 91 | 22 | 0.300 | 100 | 0.579 | 503 | 429 | 90 | 0.298 | 100 | 0.914 |
| Latin American | | | | 1703 | 1304 | 280 | 0.284 | 100 | 0.177 | 483 | 359 | 72 | 0.275 | 100 | 0.643 | 1220 | 945 | 208 | 0.287 | 100 | 0.197 |
| Native | | | | 344 | 145 | 10 | 0.165 | 100 | 0.238 | 68 | 32 | 2 | 0.176 | 100 | 0.423 | 276 | 113 | 8 | 0.162 | 100 | 0.360 |
| Total | | | | 8687 | 7060 | 1628 | 0.297 | 100 | N.A. | 1614 | 1301 | 288 | 0.293 | 100 | N.A. | 7073 | 5759 | 1340 | 0.298 | 100 | N.A. |
| Rs231362– *KCNQ1* | | | | | | | | | | | | | | | | | | | | | |
| | | | | CC | CT | TT | | | | CC | CT | TT | | | | CC | CT | TT | | | |
| South Asian | C | T | C | 1558 | 1015 | 178 | 0.751 | 99.89 | 0.464 | 100 | 79 | 12 | 0.730 | 100 | 0.488 | 1458 | 936 | 166 | 0.752 | 99.88 | 0.340 |
| East Asian | | | | 187 | 37 | 1 | 0.913 | 100 | 0.561 | 20 | 3 | 0 | 0.935 | 100 | 0.738 | 167 | 34 | 1 | 0.911 | 100 | 0.600 |
| European | | | | 2659 | 4648 | 2052 | 0.532 | 99.96 | 0.806 | 507 | 838 | 403 | 0.530 | 100 | 0.114 | 2152 | 3810 | 1649 | 0.533 | 99.95 | 0.627 |
| African | | | | 769 | 411 | 67 | 0.781 | 100 | 0.217 | 149 | 66 | 10 | 0.808 | 100 | 0.442 | 620 | 345 | 57 | 0.775 | 100 | 0.326 |
| Latin American | | | | 1279 | 1495 | 507 | 0.618 | 99.82 | 0.043 | 398 | 384 | 130 | 0.650 | 99.78 | 0.018 | 881 | 1111 | 377 | 0.606 | 99.83 | 0.392 |
| Native | | | | 203 | 227 | 69 | 0.634 | 100 | 0.664 | 46 | 43 | 13 | 0.662 | 100 | 0.556 | 157 | 184 | 56 | 0.627 | 100 | 0.859 |
| Total | | | | 6665 | 7833 | 2874 | 0.609 | 99.98 | N.A. | 1220 | 1413 | 568 | 0.602 | 99.93 | N.A. | 5435 | 6420 | 2306 | 0.610 | 99.92 | N.A. |
| Rs2283228– *KCNQ1* | | | | | | | | | | | | | | | | | | | | | |
| | | | | AA | AC | CC | | | | AA | AC | CC | | | | AA | AC | CC | | | |
| South Asian | A | C | A | 2620 | 130 | 4 | 0.975 | 100 | 0.076 | 184 | 6 | 1 | 0.979 | 100 | 0.001 | 2436 | 124 | 3 | 0.975 | 5126 | 0.280 |
| East Asian | | | | 91 | 110 | 24 | 0.649 | 100 | 0.274 | 12 | 9 | 2 | 0.717 | 100 | 0.867 | 79 | 101 | 22 | 0.641 | 404 | 0.219 |
| European | | | | 8043 | 1283 | 37 | 0.928 | 100 | 0.061 | 1500 | 239 | 9 | 0.926 | 100 | 0.878 | 6543 | 1044 | 28 | 0.928 | 15230 | 0.045 |
| African | | | | 977 | 257 | 13 | 0.887 | 100 | 0.390 | 182 | 41 | 2 | 0.900 | 100 | 0.810 | 795 | 216 | 11 | 0.884 | 2-44 | 0.385 |
| Latin | | | | 2167 | 965 | 155 | 0.806 | 100 | $4.7 \times 10^{-4}$ | 577 | 278 | 59 | 0.783 | 100 | 0.002 | 1590 | 687 | 96 | 0.815 | 4746 | 0.047 |

| | | | | | | | | | | | | | | | | | | | | | |
|---|---|---|---|---|---|---|---|---|---|---|---|---|---|---|---|---|---|---|---|---|---|
| American | | | | | | | | | | | | | | | | | | | | | |
| Native | | | | 280 | 172 | 47 | 0.733 | 100 | 0.008 | 51 | 42 | 9 | 0.706 | 100 | 0.933 | 229 | 130 | 38 | 0.741 | 794 | 0.003 |
| Total | | | | 14178 | 2917 | 280 | 0.900 | 100 | N.A. | 2506 | 615 | 82 | 0.878 | 100 | N.A. | 11672 | 2302 | 198 | 0.905 | 28344 | N.A. |
| Rs5219 – *KCNJ11* | | | | | | | | | | | | | | | | | | | | | |
| | | | | CC | CT | TT | | | | CC | CT | TT | | | | CC | CT | TT | | | |
| South Asian | C | T | T | 1158 | 1198 | 383 | 0.358 | 99.46 | 0.010 | 76 | 87 | 27 | 0.371 | 99.48 | 0.794 | 1082 | 1111 | 356 | 0.358 | 5098 | 0.010 |
| East Asian | | | | 101 | 90 | 33 | 0.348 | 99.56 | 0.086 | 9 | 10 | 3 | 0.364 | 95.65 | 0.933 | 92 | 80 | 30 | 0.347 | 40 | 0.074 |
| European | | | | 3616 | 4418 | 1317 | 0.377 | 99.87 | 0.580 | 693 | 829 | 226 | 0.366 | 100 | 0.371 | 2923 | 3589 | 1091 | 0.380 | 15206 | 0.841 |
| African | | | | 1098 | 141 | 7 | 0.062 | 99.92 | 0.290 | 197 | 28 | 0 | 0.062 | 100 | 0.320 | 901 | 113 | 7 | 0.062 | 2042 | 0.102 |
| Latin American | | | | 1375 | 1461 | 445 | 0.358 | 99.82 | 0.070 | 379 | 387 | 146 | 0.372 | 99.78 | 0.005 | 996 | 1074 | 299 | 0.353 | 4738 | 0.720 |
| Native | | | | 216 | 214 | 69 | 0.353 | 100 | 0.175 | 46 | 46 | 10 | 0.324 | 100 | 0.760 | 170 | 169 | 59 | 0.361 | 796 | 0.114 |
| Total | | | | 7564 | 7522 | 2254 | 0.347 | 99.80 | N.A. | 1400 | 1387 | 412 | 0.346 | 99.88 | N.A. | 6164 | 6135 | 1842 | 0.347 | 28282 | N.A. |
| Rs10830963 – *MTNR1B* | | | | | | | | | | | | | | | | | | | | | |
| | | | | CC | CG | GG | | | | CC | CG | GG | | | | CC | CG | GG | | | |
| South Asian | C | G | G | 933 | 1321 | 500 | 0.421 | 100 | 0.391 | 55 | 87 | 49 | 0.484 | 100 | 0.223 | 878 | 1234 | 451 | 0.417 | 100 | 0.628 |
| East Asian | | | | 76 | 108 | 41 | 0.422 | 100 | 0.808 | 8 | 15 | 0 | 0.326 | 100 | 0.020 | 68 | 93 | 41 | 0.433 | 100 | 0.375 |
| European | | | | 4527 | 3950 | 886 | 0.306 | 100 | 0.566 | 856 | 730 | 162 | 0.301 | 100 | 0.723 | 3671 | 3220 | 724 | 0.307 | 100 | 0.642 |
| African | | | | 1085 | 152 | 10 | 0.069 | 100 | 0.073 | 199 | 26 | 0 | 0.058 | 100 | 0.358 | 886 | 126 | 10 | 0.071 | 100 | 0.024 |
| Latin American | | | | 1895 | 1206 | 186 | 0.240 | 100 | 0.746 | 534 | 335 | 45 | 0.232 | 100 | 0.414 | 1361 | 871 | 141 | 0.243 | 100 | 0.916 |
| Native | | | | 296 | 167 | 36 | 0.239 | 100 | 0.070 | 57 | 38 | 7 | 0.255 | 100 | 0.846 | 239 | 129 | 29 | 0.236 | 100 | 0.052 |
| Total | | | | 8812 | 6904 | 1659 | 0.294 | 100 | N.A. | 1709 | 1231 | 263 | 0.274 | 100 | N.A. | 7103 | 5673 | 1396 | 0.299 | 100 | N.A. |
| Rs4430796 – *HNF1B* | | | | | | | | | | | | | | | | | | | | | |
| | | | | AA | AG | GG | | | | AA | AG | GG | | | | AA | AG | GG | | | |
| South Asian | A | G | G | 1324 | 1140 | 278 | 0.309 | 99.56 | 0.159 | 101 | 71 | 18 | 0.382 | 99.48 | 0.292 | 1223 | 1069 | 260 | 0.311 | 99.57 | 0.243 |
| East Asian | | | | 107 | 96 | 22 | 0.311 | 100 | 0.945 | 13 | 7 | 3 | 0.283 | 100 | 0.232 | 94 | 89 | 19 | 0.314 | 100 | 0.754 |
| European | | | | 2441 | 4661 | 2209 | 0.488 | 99.44 | 0.862 | 438 | 886 | 416 | 0.494 | 99.54 | 0.439 | 2003 | 3775 | 1793 | 0.486 | 99.42 | 0.861 |
| African | | | | 148 | 542 | 548 | 0.662 | 99.28 | 0.432 | 30 | 107 | 85 | 0.624 | 98.67 | 0.687 | 118 | 435 | 463 | 0.670 | 99.41 | 0.306 |
| Latin American | | | | 1027 | 1571 | 676 | 0.446 | 99.60 | 0.095 | 302 | 445 | 163 | 0.262 | 99.56 | 0.967 | 725 | 1126 | 513 | 0.455 | 99.62 | 0.054 |
| Native | | | | 174 | 245 | 80 | 0.406 | 100 | 0.686 | 39 | 47 | 16 | 0.387 | 100 | 0.769 | 135 | 198 | 64 | 0.411 | 100 | 0.544 |
| Total | | | | 5221 | 8255 | 3813 | 0.459 | 99.51 | N.A. | 923 | 1563 | 701 | 0.465 | 99.53 | N.A. | 4298 | 6692 | 3112 | 0.458 | 99.51 | N.A. |
| Rs12454712 – *BCL2* | | | | | | | | | | | | | | | | | | | | | |
| | | | | TT | TC | CC | | | | TT | TC | CC | | | | TT | TC | CC | | | |
| South Asian | T | C | T | 522 | 1313 | 919 | 0.428 | 100 | 0.168 | 37 | 89 | 65 | 0.427 | 100 | 0.511 | 485 | 1224 | 854 | 0.428 | 100 | 0.212 |
| East Asian | | | | 57 | 109 | 59 | 0.496 | 100 | 0.642 | 5 | 11 | 7 | 0.457 | 100 | 0.862 | 52 | 98 | 52 | 0.500 | 100 | 0.673 |
| European | | | | 3623 | 4346 | 1394 | 0.619 | 100 | 0.124 | 693 | 791 | 264 | 0.623 | 100 | 0.122 | 2930 | 3555 | 1130 | 0.618 | 100 | 0.334 |
| African | | | | 757 | 428 | 62 | 0.779 | 100 | 0.880 | 135 | 77 | 13 | 0.771 | 100 | 0.647 | 622 | 351 | 49 | 0.780 | 100 | 0.954 |
| Latin American | | | | 1399 | 1475 | 413 | 0.650 | 100 | 0.429 | 379 | 417 | 118 | 0.643 | 100 | 0.844 | 1020 | 1058 | 295 | 0.653 | 100 | 0.422 |
| Native | | | | 169 | 236 | 94 | 0.575 | 100 | 0.471 | 47 | 40 | 15 | 0.657 | 100 | 0.189 | 122 | 196 | 79 | 0.554 | 100 | 0.986 |
| Total | | | | 6527 | 7907 | 2941 | 0.619 | 100 | N.A. | 1296 | 1425 | 482 | 0.627 | 100 | N.A. | 5231 | 6482 | 2459 | 0.598 | 100 | N.A. |
| Rs16996148– *GATAD2A* | | | | | | | s | | | | | | | | | | | | | | |
| | | | | GG | GT | TT | | | | GG | GT | TT | | | | GG | GT | TT | | | |

| | | | | | | | | | | | | | | | | | | | | | |
|---|---|---|---|---|---|---|---|---|---|---|---|---|---|---|---|---|---|---|---|---|---|
| South Asian | G | T | T | 2105 | 592 | 54 | 0.127 | 99.89 | 0.104 | 139 | 49 | 2 | 0.139 | 99.48 | 0.305 | 1966 | 543 | 52 | 0.126 | 5122 | 0.046 |
| East Asian | | | | 190 | 31 | 4 | 0.087 | 100 | 0.052 | 22 | 1 | 0 | 0.022 | 100 | 0.915 | 168 | 30 | 4 | 0.094 | 100 | 0.068 |
| European | | | | 7949 | 1346 | 65 | 0.079 | 99.97 | 0.332 | 1499 | 241 | 8 | 0.074 | 100 | 0.612 | 6450 | 1105 | 57 | 0.080 | 15224 | 0.202 |
| African | | | | 896 | 310 | 40 | 0.157 | 99.92 | 0.042 | 165 | 51 | 9 | 0.153 | 100 | 0.057 | 731 | 259 | 31 | 0.157 | 2042 | 0.173 |
| Latin American | | | | 2851 | 412 | 22 | 0.070 | 99.94 | 0.095 | 800 | 107 | 7 | 0.066 | 100 | 0.109 | 2051 | 305 | 15 | 0.071 | 4742 | 0.322 |
| Native | | | | 450 | 48 | 1 | 0.050 | 100 | 0.812 | 93 | 9 | 0 | 0.044 | 100 | 0.641 | 357 | 39 | 1 | 0.052 | 794 | 0.952 |
| Total | | | | 14441 | 2739 | 186 | 0.090 | 99.95 | N.A. | 2718 | 458 | 26 | 0.080 | 99.97 | N.A. | 11723 | 2281 | 160 | 0.092 | 28328 | N.A. |
| Rs8108269 – *GIPR* | | | | | | | | | | | | | | | | | | | | | |
| | | | | TT | TG | GG | | | | TT | GT | GG | | | | TT | GT | GG | | | |
| South Asian | T | G | G | 1185 | 1219 | 320 | 0.341 | 98.91 | 0.809 | 92 | 73 | 24 | 0.320 | 98.95 | 0.121 | 1093 | 1146 | 296 | 0.343 | 5070 | 0.867 |
| East Asian | | | | 80 | 95 | 48 | 0.428 | 99.11 | 0.052 | 5 | 10 | 8 | 0.565 | 100 | 0.580 | 75 | 85 | 40 | 0.413 | 99.01 | 0.082 |
| European | | | | 4459 | 3959 | 893 | 0.309 | 99.44 | 0.741 | 831 | 738 | 170 | 0.310 | 99.49 | 0.742 | 3628 | 3221 | 723 | 0.308 | 15144 | 0.835 |
| African | | | | 443 | 582 | 216 | 0.409 | 99.52 | 0.297 | 86 | 97 | 42 | 0.402 | 100 | 0.121 | 357 | 485 | 174 | 0.410 | 2032 | 0.672 |
| Latin American | | | | 1356 | 1492 | 417 | 0.356 | 99.33 | 0.835 | 359 | 416 | 132 | 0.375 | 99.23 | 0.519 | 997 | 1076 | 285 | 0.349 | 4716 | 0.839 |
| Native | | | | 132 | 258 | 105 | 0.473 | 99.20 | 0.311 | 23 | 54 | 24 | 0.505 | 99.02 | 0.485 | 109 | 204 | 81 | 0.464 | 788 | 0.418 |
| Total | | | | 7655 | 7605 | 1999 | 0.336 | 99.33 | N.A. | 1396 | 1388 | 400 | 0.344 | 99.41 | N.A. | 6259 | 6217 | 1599 | 0.334 | 28150 | N.A. |
| Rs1884614–*HNF4A* | | | | | | | | | | | | | | | | | | | | | |
| | | | | CC | CT | TT | | | | CC | CT | TT | | | | CC | CT | TT | | | |
| South Asian | C | T | T | 1231 | 1215 | 308 | 0.332 | 100 | 0.753 | 85 | 81 | 25 | 0.343 | 100 | 0.374 | 1146 | 1134 | 283 | 0.332 | 100 | 0.922 |
| East Asian | | | | 64 | 126 | 35 | 0.436 | 100 | 0.037 | 7 | 12 | 4 | 0.435 | 100 | 0.768 | 57 | 114 | 31 | 0.436 | 100 | 0.036 |
| European | | | | 6254 | 2810 | 298 | 0.182 | 99.99 | 0.413 | 1136 | 546 | 66 | 0.194 | 100 | 0.983 | 5118 | 2264 | 232 | 0.179 | 99.99 | 0.336 |
| African | | | | 1008 | 222 | 17 | 0.103 | 100 | 0.235 | 176 | 45 | 4 | 0.118 | 100 | 0.560 | 832 | 177 | 13 | 0.099 | 100 | 0.307 |
| Latin American | | | | 1444 | 1388 | 455 | 0.350 | 100 | $4.3 \times 10^{-5}$ | 392 | 394 | 128 | 0.356 | 100 | 0.073 | 1052 | 994 | 327 | 0.347 | 100 | $2.1 \times 10^{-4}$ |
| Native | | | | 174 | 212 | 111 | 0.437 | 99.60 | 0.003 | 36 | 43 | 23 | 0.436 | 100 | 0.149 | 138 | 169 | 88 | 0.437 | 99.50 | 0.010 |
| Total | | | | 10175 | 5973 | 1224 | 0.242 | 99.98 | N.A. | 1832 | 1121 | 250 | 0.253 | 100 | N.A. | 8343 | 4852 | 974 | 0.240 | 99.98 | N.A. |

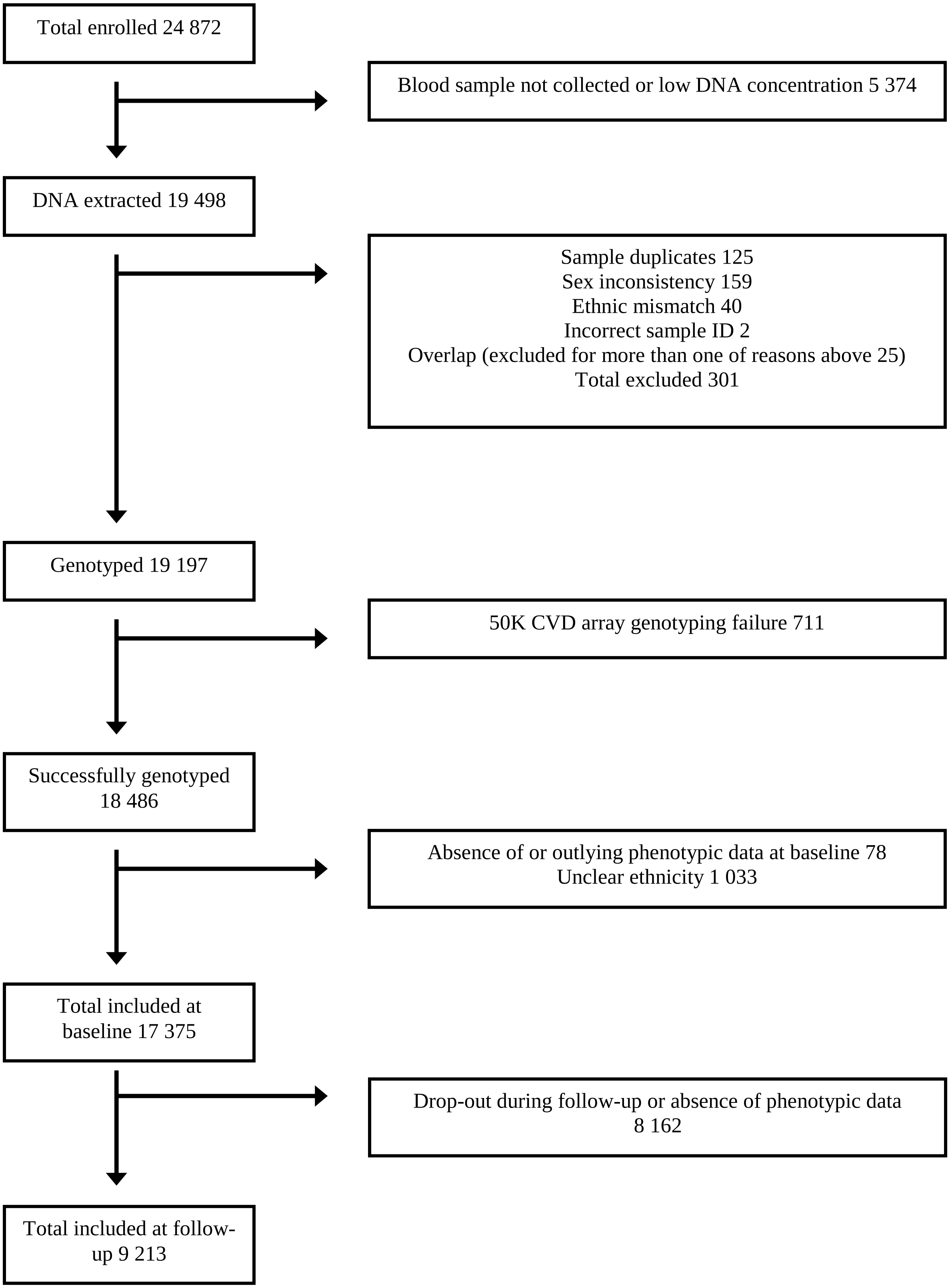


**Supplementary Figure S1.** Flow chart of the EpiDREAM study.

**Supplementary Table S2.** Distribution of depression cases and controls within each ethnicity

| Ethnicity | Depression cases (%) | Depression controls (%) |
|---|---|---|
| South Asian | 191 (6.94%) | 2563 (93.06%) |
| East Asian | 23 (10.22%) | 202 (89.78%) |
| European | 1748 (18.67%) | 7615 (81.33%) |
| African | 225 (18.04%) | 1022 (81.96%) |
| Latin American | 914 (27.81%) | 2373 (72.19%) |
| Native North American | 102 (20.4%) | 397 (79.56%) |

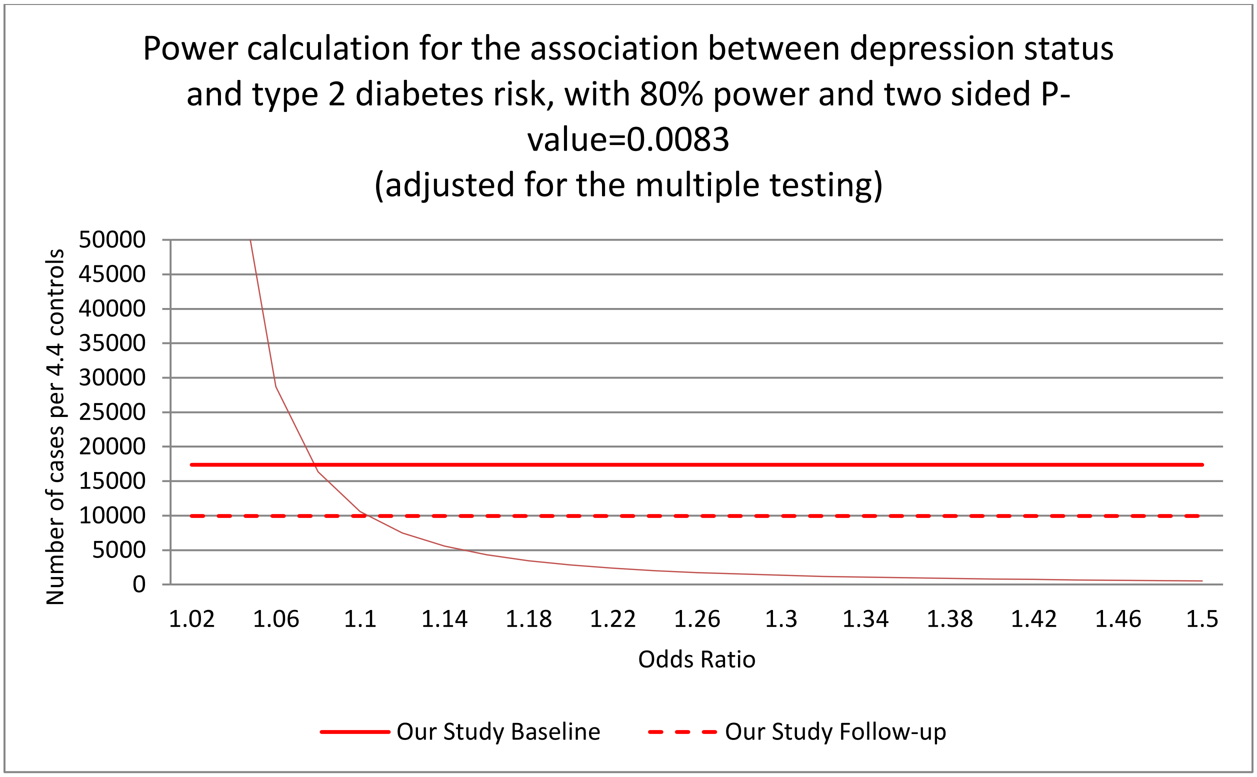


**Supplementary Figure S2.** Power calculation for association between depression and T2D risk at baseline and follow-up (adjusted for multiple testing) 2-sided *P*-value=0.0083, 80% power.

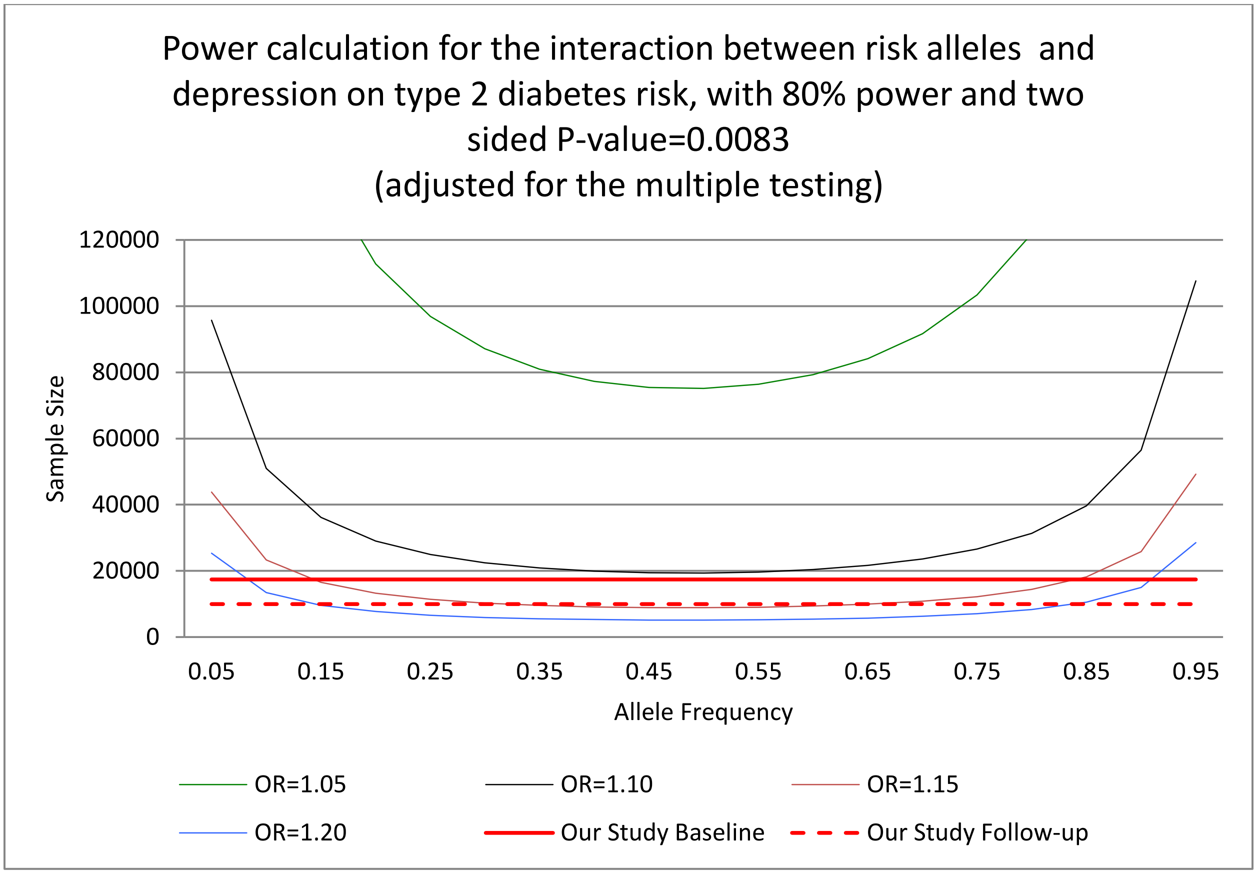


**Supplementary Figure S3.** Power calculation for interaction between risk alleles and depression on T2D risk at baseline and follow-up (adjusted for the multiple testing) 2 sided *P*-value= 0.0083.
